# The immunogenicity and safety of respiratory syncytial virus vaccines in development: A systematic review

**DOI:** 10.1111/irv.12850

**Published:** 2021-03-25

**Authors:** Jing Shan, Philip N. Britton, Catherine L. King, Robert Booy

**Affiliations:** ^1^ Anhui Provincial Children Hospital Hefei China; ^2^ The Discipline of Child and Adolescent Health Faculty of Medicine and Health The Children's Hospital Westmead Clinical School The University of Sydney Westmead NSW Australia; ^3^ Department of Infectious Diseases and Microbiology The Children's Hospital at Westmead Westmead NSW Australia; ^4^ National Centre for Immunisation Research and Surveillance (NCIRS) The Children's Hospital at Westmead Westmead NSW Australia

**Keywords:** clinical trial, respiratory syncytial virus vaccine, RSV promising vaccine, safety and immunogenicity

## Abstract

**Background:**

Respiratory syncytial virus (RSV) is a leading cause of acute lower respiratory infection globally. There are vaccine candidates in development, but a systematic review on immunogenicity and safety of vaccine is lacking.

**Methods:**

This systematic review of RSV vaccine clinical trials was undertaken using four databases. Searches were conducted using both controlled vocabulary terms such as “Respiratory Syncytial Virus, Human,” “Respiratory Syncytial Virus Infections,” “Respiratory Syncytial Virus Vaccines,” “Immunization,” “Immunization Programs” and “Vaccines” and corresponding text word terms. The included studies were limited to clinical trials published from January 2000 to 31 December 2020. RSV infection case was defined as RSV‐associated medically attended acute respiratory illness (MAARI) or RSV infection by serologically confirmed test (Western blot) during the RSV surveillance period. We calculated the relative risk of each vaccine trial with RSV infection case.

**Results:**

Of 6306 publications, 38 were included and data were extracted covering four major types of RSV vaccine candidates, these being live‐attenuated/chimeric (n = 14), recombinant‐vector (n = 6), subunit (n = 12) and nanoparticle vaccines (n = 6). For RSV infection cases, nine trials were involved and none of them showed a vaccine‐related increased MAARI during RSV surveillance season.

**Conclusion:**

LID ∆M2‐2, MEDI M2‐2, RSVcps2 and LID/∆M2‐2 /1030s (live‐attenuated) were considered the most promising vaccine candidates in infant and children. In the elderly, a nanoparticle F vaccine candidate and Ad26.RSV.preF were considered as two potential effective vaccines. A promising maternal vaccine candidate is still lacking.

## 1 INTRODUCTION

Respiratory syncytial virus (RSV) is one of the main causes of acute lower respiratory infection (ALRI) and commonly leads to pneumonia or bronchiolitis.^1^ The pattern of RSV infection in humans shows a U‐shaped age curve, with peak disease rates in those younger than 5 years and older than 65 years.^2^ A recent epidemiological study on children showed an estimated 33.1 million RSV‐ALRI episodes globally in 2015, which resulted in about 3.2 million hospitalisations; around 45% of the hospitalised patients were younger than 6 months old. The estimated annual number of deaths was 59 600 in children aged younger than 5 years, with 46% happening in children younger than 6 months.^3^ In the elderly, several studies have shown that RSV is an important cause of illness in community‐dwelling older people.^4^, ^5^ RSV may cause a similar burden of disease to non‐pandemic influenza A in older age groups.^6^ RSV is annually associated with around 177 000 hospitalisations and 14 000 deaths in US adults aged 65 years or older.^6^

In 1955, RSV was first isolated from a chimpanzee with respiratory symptoms and designated chimpanzee coryza agent. RSV is an enveloped RNA virus and belongs to the family of *Paramyxoviridae*, classified within the genus *Pneumovirus*, and it can be separated into two major subtypes, A and B. There are four important proteins on the surface of the RSV virion, which are the attachment glycoprotein (G), the fusion (F) protein, the matrix protein (M) and the small hydrophobic (SH) protein.^7^ The main human‐neutralising antibody is against the F protein which enables the virus to fuse with the membrane of respiratory cells. It is highly conserved and essential for viral viability. However, the RSV virus can make a conformational change to the F protein to avoid antibody neutralisation. In contrast, the G protein focuses on the ciliated cells of the human airway; variation of it is associated with subtype classification.^8^ Therefore, both of these two antigens have been targeted by novel vaccine candidates (and also by monoclonal antibodies). The function of M protein is thought to be in interaction with polymerised actin which destabilises cellular microfilaments to transport virion components in the host cells.^9^ However, the function of SH protein is not yet clearly known.^10^

Adverse events associated with the development of an RSV vaccine in the mid‐1960s delayed the development of an RSV vaccine for decades. At that time, a formalin‐inactivated (FI) RSV vaccine was being tested for protective efficacy. It failed due to worrying results. A large proportion of the study participants, who were exposed to natural RSV infection soon after vaccine recipients, developed enhanced respiratory disease (ERD) and unfortunately two of these children died. The subsequent investigation found that the FI vaccine did not produce neutralising antibodies and also failed to elicit CD8^+^ T cells. Instead, it induced an aggressive CD4^+^ T cell and cytokine response leading to ERD.^11^

In 2018, Mazur et al^12^ published a narrative review on RSV vaccine development. However, there has been no recent systematic review on this topic. We divided respiratory syncytial virus (RSV) vaccines under development into four major groups: particle‐based, vector‐based, live‐attenuated or chimeric, and subunit vaccines.

## 2 METHODS

### 2.1 Study objective

This study has two major aims: firstly, to systematically review the medical publications on clinical trials of RSV vaccines from 1 January 2000 to 31 December 2020 and describe immunogenicity and safety data in the published journals; secondly, to evaluate the risk of RSV infection in vaccine recipients during RSV follow‐up season.

### 2.2 Literature searches

The initial search for this systematic review of RSV vaccine clinical trials was undertaken by a medical information specialist (CK) using the following bibliographic databases: Ovid MEDLINE, Ovid EMBASE, the Cochrane Library Database of Systematic Reviews and Cochrane Central Register of Controlled Trials. Searches were conducted using both controlled vocabulary terms such as “Respiratory Syncytial Virus, Human,” “Respiratory Syncytial Virus Infections,” “Respiratory Syncytial Virus Vaccines,” “Immunization,” “Immunization Programs” and “Vaccines” and corresponding text word terms. The searches were limited to items published from 1 January 2000. The last search was conducted on 20 January 2021.

### 2.3 Screening

Items were screened using the inclusion/exclusion criteria (see Table 1) by the first author (JS). The screening was cross‐checked by the senior author (RB).

**TABLE 1:** Inclusion and exclusion criteria

Inclusion	Clinical study of RSV vaccine used in humans with a measured outcome of immunogenicity
	All ages
	English abstract and full text
	Studies published after Jan 2000 to 31 December 2020
	Human only
Exclusion	Studies with a focus on non‐vaccination prevention of RSV, for example hand washing, RSV epidemiology, treatment of RSV infection
	Animal studies

### 2.4 Data extraction

A data extraction form was developed by JS in consultation with co‐authors (RB, PB, CK). Information extracted included “title,” “name of first author,” “source,” “national clinical trials’ number (NCT),” “participants,” “vaccine candidate,” “study type,” “outcome,” and “serious adverse events.” We focused on severe prognoses and decided to limit descriptions to adverse effects that were a minimum of grade 3.^13^

### 2.5 Evaluation of data analysis

We aimed to summarise the RSV vaccine immunogenicity based on each paper's definition of “immune‐response” (described in the relevant journal papers); commonly, for instance, a ≥4‐fold rise in RSV‐neutralising antibody (NA) in seronegative children or a ≥3‐fold rise in NA in adults. Moreover, JS extracted the safety data based on the serious adverse events (SAE) presented in those papers.

The studies looked at disease prevention: a case of RSV infection was defined as RSV‐associated medically attended acute respiratory illness (MAARI) or was serologically confirmed (Western blot) during RSV surveillance season. Review Manager 5.3 was used for data analysis. A fixed‐effects model was used for data analysis, and a relative risk in vaccinated group compared with unvaccinated group with 95% confidence interval (CI) was calculated.

This review was not prospectively registered.

## 3 RESULTS

**FIGURE 1:**
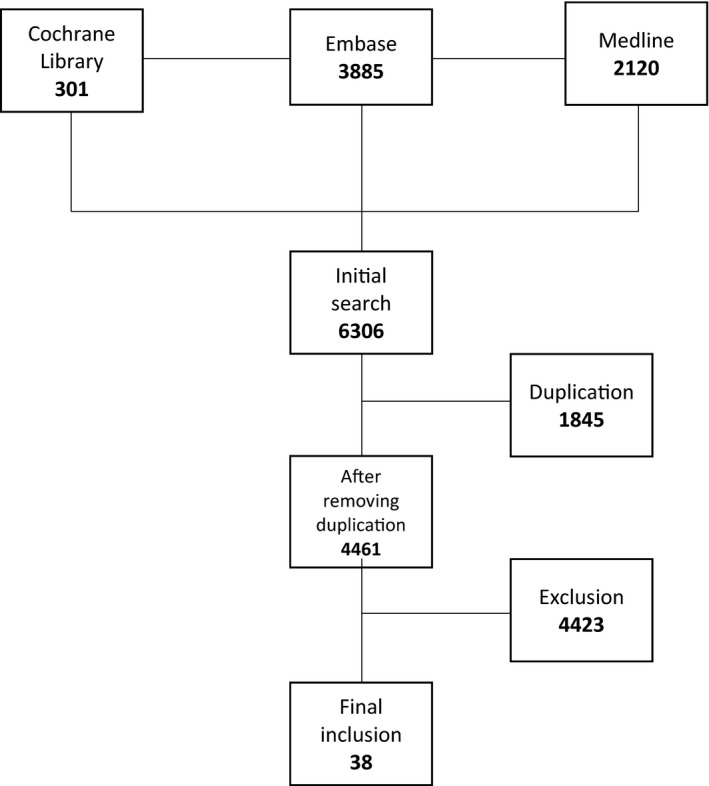
PRISMA flow chart

A total of 6306 publications were identified through the database searches. Duplicate publications and those that were not RSV vaccine clinical trials were excluded. In total, 38 publications were included covering the four major types of RSV vaccine candidates, live‐attenuated (n = 14), subunit (n = 12), vector‐based (n = 6) and nanoparticle (n = 6) (see Figure 1).

### 3.1 Live‐attenuated/chimeric vaccines

#### 3.1.1 M2‐2

The RSV M2‐2 gene mediates the transition from transcription to RNA replication, so its deletion can be used to attenuate the virus. Meanwhile, it still elicits a neutralising antibody response.^14^ In 2015, Karron et al reported a MEDI M2‐2 study on seronegative children aged 6‐24 months. The result was ≥4‐fold of neutralising antibody titres in 95% (19/20) vaccinees and a ≥4‐fold rise of anti‐F antibody in 90% (18/20) of vaccine recipients while there was no antibody rise in non‐RSV‐infected placebo recipients. Two grade 3 fever serious adverse events (SAE) were reported (NCT01459198).^15^

Furthermore, two studies (NCT02237209 and NCT02040831) explored the safety and immunogenicity of the LID ∆M2‐2 vaccine in RSV‐seronegative children aged from 6 to 24 months. LID ∆M2‐2 appeared to have acceptable infectivity and immunogenicity: 90% (18/20) of vaccine recipients had a ≥4‐fold rise in both neutralising antibody and anti‐F IgG antibody. The placebo group showed none with a 4‐fold rise in the antibody. Importantly, the subsequent RSV season surveillance showed 8 of 19 vaccinees had a ≥4‐fold increase in either neutralising antibody or anti‐F IgG titres compared to pre‐RSV season, but only in 2 of 9 placebo recipients, indicating the vaccine's anamnestic response capability.^16^ In 2019, Cunningham et al^17^ reported two studies (NCT02890381 and NCT02948127) which demonstrated RSV LIDcp∆M2‐2 vaccine candidate was overattenuated. In 2020, McFarland et al reported two studies (NCT02952339 and NCT02794870), which analysed the safety and immunogenicity of the LID/∆M2‐2 /1030s vaccine in 33 RSV‐seronegative children aged from 6‐24 months. The results showed serum RSV‐neutralising antibody and anti‐RSV IgG increased ≥4‐fold in 95% and 100% of vaccinees, respectively. No serious adverse events were reported. In the follow‐up RSV season, RSV‐MAARI were found in 1 vaccinee vs 2 placebo recipients. Thus, this vaccine candidate was promising in children.^18^ McFarland et al reported on two studies (NCT03102034 and NCT03099291). These revealed the D46/NS2/N/"M2‐2‐HindIII vaccine candidate was highly immunogenic in RSV‐seronegative children aged from 6‐24 months, and further study was warranted.^19^

#### 3.1.2 RSVcpts

Cold‐passage (cp) mutagenesis is based on an alteration to render the virus temperature‐sensitive (ts) so that it can only replicate in the upper respiratory tract, not in the lungs. Therefore, it is used in vaccine development.^20^, ^21^

The 404, 248 and 1030 mutations are considered as the main attenuated genotypes determining mutation.^22^ RSV cpts‐248/404 vaccine is a lineage of RSVcpts vaccine product, which has been studied in infants and children.^23^, ^24^, ^25^ RSV cpts‐248/404 appeared to increase upper respiratory tract congestion in 1‐ to 2‐month‐old infants in a double‐blind RCT. Because of concern regarding pathogenicity of this vaccine virus, cpts‐248/404 needs more attenuation for infants’ use.^26^

#### 3.1.3 SH

The SH gene has been variously deleted to produce live‐attenuated vaccine candidates. The function of this gene is not yet known.^10^ Due to only 44% of infants in the two‐dose group vs no infants in the placebo group having a ≥4‐fold antibody rise in a double‐blind RCT, rA2cp248/404/1030 ΔSH needs further refinement regarding immunogenicity. No vaccine‐related serious adverse event was reported.^27^

MEDI‐599 is another SH deletion vaccine. Unfortunately, it showed increased medical attendance due to lower respiratory infection in vaccinated children in a phase 1 double‐blind RCT (NCT00767416); hence, further study of its safety profile is needed.^28^

Cold‐passage/stabilised 2 (RSVcps2) is produced from MEDI‐599 with adding stabilised 248 and 1030 mutations. In 2018, Buchholz et al^22^ reported a phase 1, RCT conducted in RSV‐seronegative children aged from 6 to 24 months. It showed that a ≥4‐fold neutralising antibody rise was seen in 59% of the vaccine group vs 13% in the placebo group. Furthermore, a ≥4‐fold anti‐F IgG antibody rise occurred in 68% of vaccinees vs 13% in the placebo group. However, the same rate (50%) of respiratory tract infection and febrile events were in both the vaccine and placebo group. Moreover, one serious adverse event in the vaccine group was posted.^22^

#### 3.1.4 NS

RSV/NS2/1313/I1314L is a vaccine candidate which contains two attenuating elements: one is NS2 gene and the other is codon 1313 of the RSV polymerase gene and the stabilising missense mutation I1314L. In 2020, Karon et al reported a Phase 1, RCT (NCT01893554) which demonstrated this vaccine candidate was well‐tolerated and immunogenic in RSV‐seronegative children aged 12‐59 months and it primed for anamnestic responses after RSV natural exposure. During the follow‐up RSV season, 4 of 20 vaccinees vs 3 of 10 placebo recipients had RSV‐MAARI. Therefore, the further evaluations were warranted.^29^

#### 3.1.5 G

RSV ΔG is a live‐attenuated vaccine candidate which is deleted G attachment protein. In 2020, Verdijk et al posted a first‐in‐human RCT which recruited 48 healthy adults aged from 18 to 50 years. RSV ΔG was well tolerated without any clinical concerns; however, there was no significant induction of an immune response. Therefore, a further study about its safety and immunogenicity in children is needed.^30^

#### 3.1.6 MEDI‐534

MEDI‐534 is a vaccine candidate using a parainfluenza virus type 3 (PIV3) backbone genome, which was altered to express RSV F protein.^31^ Three RCTs have been conducted to evaluate the safety and immunogenicity in infants and children. In 2004, Belshe et al published the results of a Phase 1, double‐blind RCT trial: 95% of children in the vaccine group had a ≥4‐fold RSV‐neutralising antibody rise while no placebo recipient had a similar rise. There was also evidence for antibody elicitation against PIV3. This study supported the further study of MEDI‐534.^32^ Gomez et al reported a Phase 1, double‐blind RCT; it showed this vaccine‐induced minimal immune responses in RSV‐seropositive children aged 1 to 9 years (NCT00345670). There was no significant difference in the side effect event rates between the vaccine and placebo groups.^33^ Thirdly, a Phase 1, double‐blind RCT was conducted in 49 RSV/PIV3 seronegative children aged 6‐24 months. The results were better in those given multiple doses (ie 2 or 3) and at a higher dose median tissue culture infective dose (TCID50), dosage of 10^6^, but even then only about 50% responded with a ≥4‐fold neutralising antibody rise so it was not a strong candidate; only one of 17 in the placebo group had a ≥4‐fold rise in neutralising antibody likely due to a wild‐type RSV infection. Also, a favourable immune response to PIV3 was observed. There was no serious adverse event.^34^

### 3.2 Subunit vaccines

#### 3.2.1 BBG2Na

The BBG2Na is a subunit vaccine candidate purified from a prokaryote‐expressed protein (in *Escherichia coli*). A single‐blind RCT in younger adults from 2001 showed that the 100ug and 300ug vaccine groups had greater immune response than the 10ug group with 33%‐71% developing a virus‐neutralising response; only 7% had this response in the placebo group. Giving a second or third dose did not provide a significant booster response. Most recipients of 100 µg or 300 µg vaccines had at least 4‐fold rise in antibody measured in G2Na‐specific ELISA units. No serious adverse event was reported.^35^ There appears to be no follow‐up human only study on this product published since 2001.

#### 3.2.2 Prefusion vaccines (RSV Pre F, RSV F‐020 and RSV F‐024)

RSV‐F is subject to conformational alteration during fusion of the virus with human cells—the prefusion structure exposes more antigenic sites for neutralising antibody than the post‐fusion structure. The recombinant RSV prefusion protein F vaccine was purified in Chinese hamster ovary cells and manipulated to retain the prefusion conformation.^36^, ^37^, ^38^

RSV Pre F was evaluated through a recent RCT in healthy young men. The results showed all vaccine recipients achieved ≥1:512 RSV A‐neutralising antibody titre by day 30 with a 3.2‐ to 4.9‐fold rise in titres. Antibody responses remained high until day 60. No vaccine‐related serious adverse event was noted.^38^ These results supported further research.

Two RCTs were conducted with the F‐020 and F‐024 products to investigate the safety and immunogenicity in non‐pregnant women aged 18‐45 years. All RSV vaccine groups exhibited a rise in RSV‐A‐neutralising antibody (NA) of 3.1‐ to 3.9‐fold, while the control group showed no increase. Furthermore, all RSV vaccine groups achieved a >14‐fold palivizumab competitive antibody (PCA) concentrations on day 30 that then waned but still was above baseline on day 90. In the control group, only 6% or fewer recipients had an NA immune response (days 30, 60 and 90). F‐020 and F‐024 recipients had a similar safety profile to the control group recipients, and no SAEs were considered vaccine‐related (NCT02360475, NCT02753413).^39^

#### 3.2.3 Post‐fusion F

MEDI7510 is a post‐fusion (post‐F) protein vaccine candidate that has been evaluated with or without an adjuvant, an analogue of monophosphoryl lipid A called glucopyranosyl lipid A (GLA), which is a toll‐like receptor‐4 (TLR‐4) agonist. Three RSV post‐F trials also have been performed in adults aged ≥60 years. The first, a Phase 1a, double‐blind, RCT (NCT02115815), tested its immunogenicity and safety. It showed 50% of participants in the dosage of 80ug with adjuvant group had a ≥3‐fold geometric mean fold rise in microneutralisation. All vaccinees in this group also had a ≥3‐fold rise in anti‐F IgG antibody and PCA. Conversely, no such rises were found in the placebo group.^40^

A Phase 2b, RCT was recently performed in almost 2000 participants aged at least 60 years to prevent elderly vaccinees against developing RSV illness. It was unsuccessful showing a vaccine efficacy of −7.1% (NCT02508194).^41^ A third study, also published in 2017, with the elderly, was a Phase 1, double‐blind, RCT (NCT02289820). The vaccinees receiving a 120 µg vaccine dose, with 5.0 µg GLA adjuvant, had the highest frequency of a ≥3 geometric mean fold rise in anti‐F IgG antibody. No controls had such a response. Similar reactogenicity and side effects were observed in the intervention and control groups.^42^

In 2019, a first‐in‐human RCT (NCT02298179) was reported by Leroux‐Roles et al. This study examined the safety and immunogenicity of an engineered recombinant RSV fusion glycoprotein in its post‐fusion conformation (RSV F subunit vaccine) in healthy non‐pregnant women and men aged 18‐45 years. The vaccine was well‐tolerated and the enhanced immune responses could last through 6 months of follow‐up. However, no booster effect was found after the second dose.^43^

#### 3.2.4 RSV‐A vaccine with subunit F, G and M

A subunit vaccine which contained purified RSV A proteins F, G and M was initially developed over a decade ago. In 2008, Falsey et al examined this vaccine candidate in 1169 older people ≥65 years with high‐risk factors (eg congestive heart failure and chronic obstructive pulmonary disease) to compare the immunogenicity and safety with trivalent influenza vaccine in a Phase 2 RCT. A total of 400 participants received this vaccine candidate with adjuvant; 383 received the vaccine without adjuvant, and 386 were in the placebo group. All the participants were given trivalent influenza vaccine. The results showed no interference between RSV vaccinations and trivalent influenza vaccination; furthermore, 129 of 392 participants achieved a ≥4‐fold rise in neutralising antibody rise in the adjuvant group; 168 of 378 participants had a ≥4‐fold rise in neutralising antibody rise in the non‐adjuvant group. Only 3 of 380 had such a rise in the placebo group. Only one vaccine‐related serious adverse event occurred in the non‐adjuvant group. In comparison to placebo, this vaccine candidate did not increase RSV infection in the RSV surveillance seasons. The results of this trial supported its further study in the elderly.^44^

Then, a Phase 1 RCT enrolled 561 healthy people aged ≥65 years, which studied the same recombinant subunit vaccine (containing F, G and M), to assess NA levels and the levels of RSV F‐specific and RSV G‐specific antibodies. Only the unadjuvanted 100 µg product induced a minimum of 50% recipients to have a ≥4‐fold rise in NA against RSV‐A; meanwhile, it showed that neutralising antibody rise can provide cross‐protection against RSV‐B. There was no overall antibody increase in the placebo group and no vaccine‐related serious side event.^45^

#### 3.2.5 RSV‐A vaccine with subunit SHe

In 2018, Langley et al reported a first‐in‐human RCT about the safety and immunogenicity of a novel synthetic RSV antigen based on the ectodomain of the small hydrophobic glycoprotein (SHe) of RSV subgroup A, formulated with either the lipid and oil‐based vaccine platform DepoVax (DPX‐RSV[A]) or alum (RSV[A]‐Alum), in healthy adults aged 50‐64 years. The vaccinees, who randomly received two levels (10 µg and 25 µg) of SHe with each formulation, were compared to the placebo group. A booster was given to the vaccinees on Day 56. Robust immune responses were observed in the DPX‐RSV(A) 10 µg and 25 µg groups, lasting until Day 421 in the DPX‐RSV(A) 25 µg group. No serious adverse event was found.^46^

#### 3.2.6 PFP (purified F protein)

Two purified F protein vaccine candidates were reported on in 2003. One, an RSV purified fusion protein 2 (PFP‐2) subunit vaccine, was tested in a Phase 1, RCT to determine safety and immunogenicity in 35 women in their third trimesters of pregnancy and their subsequently born children. 95% of vaccine recipients had a ≥4‐fold rise in anti‐F IgG antibodies. Further, geometric mean concentrations (GMC) of RSV anti‐F IgG antibodies in vaccine recipients’ infants at birth, 2 and 6 months after delivery were 4‐fold higher than those in infants of the placebo group, respectively. There was no safety concern.^47^

In a related study, a Phase 2, adjuvant‐controlled trial on purified fusion protein‐3 (PFP‐3) vaccine determined immunogenicity in 294 RSV‐seropositive children with cystic fibrosis. Compared to the placebo group, the vaccine group had significant ≥4‐fold titre rises in RSV‐neutralising antibody A (67% vs 2%), RSV‐neutralising antibody B (55% vs 3%) and anti‐F IgG (97% vs 1%) at day 28. Furthermore, antibody in the vaccine group remained elevated while declining somewhat through the RSV season.^48^

### 3.3 Vector‐based vaccines

#### 3.3.1 MVA‐RSV and PanAd3‐RSV

The RSV vector‐based vaccines contain inserted portions of RSV protein‐encoding genome using either an innocuous adenovirus or another non‐pathogenic virus vector‐like modified vaccine Ankara (MVA).^49^ They are hypothesised to have the advantage of increased mucosal IgA and cellular immune responses.^50^

MVA and Simian adenovirus (PanAd3) are vectors of RSV vaccines (MVA‐RSV and PanAd3‐RSV) both used to encode RSV protein F, N and M2‐1. In 2015, a Phase 1, open‐label, RCT, enrolled 42 healthy adults aged 18‐50 years. The primary PanAd3‐RSV vaccines were given through intranasal (IN) spray in two groups and intramuscular (IM) injection in the other two groups. The booster, PanAd3‐RSV or MAV‐RSV, was administrated by IM. RSV‐neutralising antibody increased in the primary PanAd3‐RSV IM group after the first dose and in the primary IN groups but only after the IM booster. A higher anti‐F IgG rise was observed in 19 of 19 participants in the primary IM groups while a rise was seen in 8 of 17 in the IN groups. After boosting, the participants in the IN groups achieved a similar anti‐F IgG rise to the ones in the primary IM groups. No vaccine‐related serious adverse event occurred.^51^

In 2020, Samy et al reported a Phase 1 clinical trial evaluating the safety and immunogenicity of MVA‐BN‐RSV in two age groups: 18‐49 years and 50‐65, respectively. No serious adverse event was observed and no difference of safety and immunogenicity was found between the two age groups. Therefore, a further trial in an older adult population was warranted.^52^

#### 3.3.2 rBCG‐N‐hRSV

In 2020, Abarca et al published a Phase 1, RCT (NCT03213405) in healthy males aged 18‐50 years evaluating the safety, tolerability and immunogenicity of a recombinant Mycobacterium bovis BCG vaccine expressing the nucleoprotein of RSV (rBCG‐N‐hRSV). No vaccine‐related serious adverse event was recorded and the serum IgG increased enough to neutralise RSV in vitro.^53^

#### 3.3.3 ChAd155‐RSV

ChAd155‐RSV is a vaccine using the viral vector chimpanzee‐adenovirus‐155, encoding RSV fusion (F), nucleocapsid and transcription antitermination proteins. In 2020, Cicconi et al reported a first‐in‐human, Phase 1, RCT (NCT02491463), in healthy adults aged 18‐45 years. This study assessed the safety and immunogenicity of ChAd155‐RSV. No serious adverse event was observed and ChAd155‐RSV generated an increase to RSV specific humoral and cellular immune responses.^54^

#### 3.3.4 Ad26.RSV preF

Ad26.RSV.preF is an RSV vaccine candidate with an adenovirus serotype 26 (Ad26) vector encoding RSV F protein stabilised in its prefusion conformation (pre‐F). Two relevant trials have been published. One is a Phase 1, clinical RCT (NCT02926430) including 72 healthy older adults ≥60 years. No serious vaccine‐related adverse events were observed and both RSV‐specific humoral and cellular immune responses were elicited.^55^ A Phase 2a, RCT (NCT03339713) evaluating the safety and immunogenicity of Ad26.RSV.preF plus an influenza vaccine in 180 healthy adults ≥60 years, was conducted in which Ad26.RSV.preF and Fluarix were randomly given to vaccinees. Coadministration of the two vaccines was well tolerated, and no vaccine‐related serious adverse event was found. Furthermore, both RSV and influenza‐specific immune responses were elicited.^56^

### 3.4 Nanoparticle vaccines

#### 3.4.1 Nanoparticle F

A Phase 1, observer‐blind, RCT (NCT01290419) was conducted in 150 healthy adults aged 18‐49 years. All vaccinees developed a 7‐ to 19‐fold increase in anti‐F IgG antibody and a 7‐ to 24‐fold increase in PCA. Furthermore, from 7.7% to 44.4% of participants in the vaccine groups had a ≥4‐fold rise in the RSV A and B microneutralising antibody. In the placebo group, these antibody levels were at or near the baseline. No serious vaccine‐related adverse event occurred.^57^

Two Phase 2 trials were conducted in women aged from 18 to 35 years. In 2016, Glenn et al reported on an observer‐blind, RCT (NCT01704365) in 330 healthy non‐pregnant women of childbearing age. The results showed a 6.5‐ to 16.5‐fold anti‐F IgG rise after 2‐dose alum adjuvanted vaccines; moreover, there was a 2.7‐ to 3.5‐fold rise in RSV/A and B‐neutralising antibodies, with no significant rise in these antibodies in the placebo group. No serious vaccine‐related event was reported.^58^ Another phase 2, observer‐blind, RCT (NCT01960686) in 2017, enrolled 720 healthy women. Approximately 90% of vaccinees in either the single‐dose 120ug (0.2 mg and 0.4 mg alum) groups or the two‐dose of 60 µg groups developed anti‐F IgG seroconversion (ie ≥4‐fold anti‐F IgG antibody rise). Similarly, more than 95% of vaccinees achieved a seroconversion in PCA. A strong immune response in the one‐dose vaccine recipients resulted in serological evidence of a halving in RSV infection reduction from Day 0 through 90. In addition, the antibody response in the one‐dose 120 µg with 0.4 mg alum adjuvant was evidenced from day 14 to day 90. No serious adverse event was found.^59^ In 2020, Madhi et al reported a Phase 3, RCT evaluating the safety and immunogenicity of RSV F nanoparticle vaccine in pregnant women and vaccine efficacy against RSV‐associated lower respiratory tract infection in their infants. Pregnant women (n = 4636) were randomly recruited into two groups, and their 4579 live infants were included. During the first 90 days of the trial in infants, the vaccine efficacy (VE) of RSV‐associated medically significant lower respiratory tract infection was 39.4%. The VE of RSV‐associated lower respiratory tract infection with severe hypoxemia was 48.3%, and the VE of RSV‐associated hospitalisation was 44.4%. The percentage of serious adverse events in recruited women was similar among the two groups. Although the result did not meet the prespecified success criterion for efficacy against RSV‐associated medically significant lower respiratory tract infection in infants up to 90 days of life, the results with respect to the other end points of RSV‐associated disease in infants still warranted a further study on potential benefits of maternal RSV vaccination.^60^

One trial was conducted in older adults. In 2017, Louis Fries et al conducted a Phase 1, observer‐blind, RCT (NCT01709019), which involved 220 healthy adults ≥60 years without cardiopulmonary issues. Two dosages (60 µg and 90 µg) of vaccine with/without alum adjuvant were given to the vaccinees, respectively. Meanwhile, trivalent influenza vaccine (TIV) was given in all the vaccine and placebo groups. This nanoparticle vaccine trial reported a 3.6‐ to 5.6‐fold anti‐F IgG rise was observed in the group of 60 µg dose of vaccine with adjuvant and the response persisted until 12 months after vaccination. Furthermore, the PCA response was parallel to the anti‐F antibody response. Three subjects in the placebo group had a ≥4‐fold neutralising antibody rise; this was considered as due to wild RSV exposure. There was no interaction between RSV nanoparticle F vaccine candidates and TIV, and no vaccine‐related serious event occurred.^61^

#### 3.4.2 mRNA RSV F nanoparticle

In 2020, Aliprantis et al reported a randomised, placebo‐controlled study, evaluating the safety and immunogenicity of an mRNA‐based RSV F nanoparticle vaccine (mRNA‐1777) in healthy young adults aged 18‐49 years and older adults aged 60‐79 years. RSV F antibody increased in both the young and older age groups, and there was no serious adverse event. Thus, a further study for mRNA‐1777 in vulnerable populations is warranted.^62^

## 4 RSV INFECTION CASES

### 4.1 RSV Infection in the live‐attenuated vaccine candidates

The relevant data were pooled from LID∆M2‐2, MEDI M2‐2, RSVcps2, LID/∆M2‐2/1030s and RSV/NS2/1313/I1314L.^15^, ^18^, ^22^, ^29^, ^63^ All these candidates were live‐attenuated vaccine candidates examined in young children. Moreover, each trial had RSV season follow‐up. RSV‐associated medically attended acute respiratory illness (MAARI) cases were detected during the RSV surveillance periods. Due to study differences, meta‐analysis was not possible. However, these data did not show a significant rate of reduction (Table 2).

**TABLE 2:** MAARI in live‐attenuated vaccine trials

Tile	Target population	Vaccine candidate	Number of participants with RSV‐associated medical attendant acute respiratory illness during RSV season in the vaccine group	Number of participants with RSV‐associated medical attendant acute respiratory illness during RSV season in the placebo group	Relative risk	Dosage in plaque‐forming unit (PFU)
Live‐attenuated respiratory syncytial virus candidate with deletion of RNA synthesis regulatory protein M2‐2 is highly immunogenic in children^63^	RSV‐seronegative children from 6‐24 mo	LID ∆M2‐2	0 of 20	1 of 9		10^5^
A gene deletion that up‐regulates viral gene expression yields an attenuated RSV vaccine with improved antibody response in children^15^	RSV‐seronegative children aged 6 to 24 mo	MEDI M2‐2	1 of 20	2 of 10	0.25 95%, CI 0.03‐2.44	10^5^
Live respiratory syncytial virus (RSV) vaccine candidate containing stabilized temperature sensitivity mutations is high attenuated in RSV‐seronegative infants and children^22^	RSV‐seronegative children aged 6‐24 mo	RSVcold‐passage/stabilised 2 (RSVcps2)	3 of 34	2 of 16	0.71 95%, CI 0.13‐3.82	10^5.3^
Respiratory syncytial virus attenuated by M2‐2 deletion and stabilized temperature sensitivity mutation 1030s is a promising vaccine candidate in children^20^	RSV‐seronegative children aged 6‐24 mo	LID/∆M2‐2 /1030s	1 in 20	2 in 11	0.28 95%, CI 0.03‐2.70	10^5^
Safety and immunogenicity of the respiratory syncytial virus vaccine RSV/DELTANS2/DELTA1313/I1314L in RSV‐seronegative children^31^	RSV‐seronegative children aged 12‐59 mo	RSV/NS2/1313/I1314L	4 in 20	3 in 10	0.67 95%, CI 0.18‐2.42	10^6^

Abbreviation: MAARI, medically attended acute respiratory illness.

### 4.2 RSV infection in the subunit vaccine candidates confirmed by western blot during RSV season

Four trials were found, of which two of them were subunit vaccines while the other two were nanoparticle vaccines. The subunit vaccine candidates were PFP‐3 and PFP‐2. The data were collected in the children aged from 1 to 12 years in the PFP‐3 study, and the infants with maternal vaccination in the PFP‐2 trial.^47^, ^64^ The relative risks of RSV infection between the vaccine and placebo groups were 0.82 (95%, CI 0.56‐1.22) and 0.19 (95%, CI 0.02‐1.51), respectively. There was no significant reduction of RSV infection (Table 3).

**TABLE 3:** RSV infection cases in subunit and nanoparticle vaccine candidates

Title	Target population	Vaccine candidate	Number of participants with RSV infection during RSV season in the vaccine group	Number of participants with RSV infection during RSV season in the placebo/control group	Relative risk	Comments
Immunogenicity of a new purified fusion protein vaccine to respiratory syncytial virus: a multi‐center trial in children with cystic fibrosis^64^	RSV‐seropositive children with CF aged 1‐12 y	PFP‐3 subunit	33 in 130	41 in 133	0.82 95%, CI 0.56‐1.22	Control group: alum adjuvant
Safety and immunogenicity of respiratory syncytial virus purified fusion protein‐2 vaccine in pregnant women^47^	healthy women in the third trimester of pregnancy and their offspring	PFP‐2 subunit	1 in 20	4 in 15 (placebo group)	0.19 95%, CI 0.02‐1.51	This result is about the infants’ follow‐up during their first RSV season.
A randomized, blinded, controlled, dose‐ranging study of a respiratory syncytial virus recombinant fusion (F) nanoparticle vaccine in healthy women of childbearing age^58^	18‐ to 35‐year older non‐pregnant and non‐lactating healthy women.	RSV‐F nanoparticle vaccine	26 in 244	12 in 56	0.48 95%, CI 0.29‐0.80	The data from vaccinees with 1 or 2 doses (60 µg or 90 µg) with/without alum adjutant
A phase 2 randomised, observer‐blind, placebo‐controlled, dose‐ranging trial of aluminium‐adjuvant respiratory syncytial virus F particle vaccine formulation in healthy women of childbearing age^59^	18‐ to 35‐y healthy women	RSV‐F nanoparticle vaccine	36 in 352	18 in 84	0.50 95%, CI 0.27‐0.92	The data of vaccinees with one‐dose groups (120 µg or 60 µg)

### 4.3 RSV infection in nanoparticle vaccine candidates confirmed by western blot during RSV season

Two RSV‐F nanoparticle vaccine trials^58^, ^59^ were conducted in healthy women of childbearing age. The two relative risks were similar; 0.48 (95%, CI 0.29‐0.80) was from all active vaccinees compared to placebo recipients, while 0.50 (95%, CI 0.27‐0.92) was from pooled one‐dose (120 µg, 60 µg) vaccinees compared to placebo recipients. A vaccine protective effect was revealed according to the relative risk results (Table 3).

## 5 DISCUSSION

RSV is deemed to be one of the most important public health care issues in young children. The World Health Organization (WHO) has predicted an effective RSV vaccine will come in the next 5‐10 years. This systematic review covered 4 major groups of vaccine candidates which are under development: live‐attenuated, subunit, recombinant and nanoparticle. These vaccine candidates are targeting several populations: infants and young children, elderly and pregnant women (or women of maternal age).

In infants and children, the age of most concern is the first 6 months of their life; although they have some maternal immune protection, the risk of severe RSV infection is still high.^65^ Many children ≥6 months are RSV‐naive, and they are similar to infants in less than 6 months, except with a more mature immune system.^66^ Almost all live‐attenuated vaccine trials were in infants older than 6 months— they are a proxy for younger infants. Maternal vaccination is one of the best strategies for protection against RSV and avoiding ERD in infants. Ideally, boosting maternal RSV antibody level from at least 3 months prior to labour could make antibodies available for trans‐placental transfer.^67^ In this review, only PFP‐2 study was conducted in pregnant women and their offspring. However, there was no further research on this candidate since 2003.

Another difficulty is balancing vaccine attenuation and immunogenicity: either under‐attenuation causing more side effects or over‐attenuation reducing vaccine infectivity. Both are problematic for optimal vaccine development.^68^ LID ∆M2‐2, MEDI M2‐2 and LID/∆M2‐2 /1030s trials showed encouraging immunogenicity results. All of them induced at least 4‐fold antibody rise in both neutralising antibody and anti‐F IgG antibody in 90% of vaccinees. These robust immune responses showed the potential of protection against RSV exposure. Although there were serious events in the trials, there was not a statistically significant difference to their placebo groups. RSVcps2 had less side effect than MEDI‐599, and also, it induced a favourable immune response in NA and anti‐F antibody. According to the MAARI rate in the subsequent RSV season, LID ∆M2‐2, MEDI M2‐2, RSVcps2 and LID/∆M2‐2 /1030s did not cause an ERD case. Due to the side effects of RSV cpts‐284/404, further attenuation has been proposed in the cold‐passage temperature live‐attenuated vaccine. This could guide future vaccine development. Although the subunit vaccine candidate PFP‐3 had encouraging immunogenicity and safety profile in children with cystic fibrosis, it is clearly not considered as a promising vaccine because no significant reduction of RSV infection was observed in the following RSV season.^69^ In summary, LID ∆M2‐2, MEDI M2‐2, RSVcps2 and LID/∆M2‐2 /1030s are the promising live‐attenuated vaccine candidates in the future.

RSV infection has caused a serious burden of disease in the elderly as age‐related changes cause a weakening of the immune system.^70^ Therefore, a potent antigen, adjuvant use or high dose given should be preferred in relevant vaccine products.^71^ MEDI‐7510, RSV vaccine A with subunit F, G, M proteins, Ad26.RSV.preF, and nanoparticle F vaccine candidates were conducted in older adults. In addition, the unpublished trial for BBG2Na also involved older people.^72^

Two trials of Ad26.RSv.preF showed encouraging results of safety and immunogenicity in the elderly. However, MEDI‐7510 failed to protect against RSV illness in a Phase 2b trial with almost 2000 participants, although the other two vaccines (subunit and the nanoparticle) demonstrated much better results. Hence, MEDI‐7510 was not recommended for further study. Although the RSV‐A vaccine with subunit F, G and M proteins, conducted in the elderly, had acceptable results regarding safety and immunogenicity, it does not appear to have been advanced further with no follow‐up trials found in 10 years of subsequent literature. Moreover, a human and animal mixed trial for BBG2Na was published. The results showed this subunit vaccine was safe and immunogenic to the vaccinees (aged 60‐80 years).^73^ However, an unpublished relevant Phase 3 trial for this candidate failed to prove safety in the elderly due to the vaccine‐related side adverse events.^72^

The 60 µg of nanoparticle F vaccine candidate with adjuvant had a favourable immune response and its persistence was long enough to cover a whole RSV season. Therefore, nanoparticle F and Ad26.RSV.preF could be thought as the promising vaccine candidate for older people.

Another two subunit candidates (F‐020 and F‐024) and a nanoparticle F vaccine candidate were conducted in women of childbearing age. All of them showed a 3‐month‐rise of immune antibody, which demonstrated the possibility of placental antibody transportation in the future. F‐024 had less vaccination local reaction than Tdap, suggesting F‐024 could be more suitable in pregnant women due to less pain from injection. Results of the relative risks of RSV infection cases in the surveillance seasons showed an acceptable protective effect for one‐dose nanoparticle candidate given in healthy women of childbearing age. Moreover, the single‐dose 120 µg RSV F protein vaccine with 0.4 mg adjuvant was timely and strongly immunogenic. Similar immunogenicity effects for the nanoparticle F vaccine candidate were observed between the one and two doses groups with adjuvant. In fact, one dose is more convenient and still gives a strong antibody response in women of childbearing age. It was examined in a Phase 3 trial (NCT02624947) in pregnant women in their third trimesters.^59^ However, the result did not meet the expected success criterion; therefore, we still lack a promising maternal vaccination.

Limitations of this systematic review include that it is limited to publications in English only and was date limited from January 2000 to avoid duplicating the results of an earlier review.^74^ Also, this review presented little information about vaccine‐induced cellular immune response due to lack of relevant data. A meta‐analysis on RSV vaccine candidates was not undertaken due to the heterogeneous nature of the vaccine candidates and targeting populations.

## 6 CONCLUSION

RSV infection is one of the most common causes of acute upper respiratory tract infection, which primarily affects children and the elderly. There is currently no licensed RSV vaccine. Recently, a number of studies have been conducted using RSV vaccines, with some encouraging results.

Live‐attenuated vaccines target infants and children mostly while the vaccines of the other three types (subunit vaccine, the vector‐based, and nanoparticle vaccine) primarily focus on maternal and elderly vaccination.

Encouraging results in both vaccine immunogenicity and safety were illustrated. LID ∆M2‐2, MEDI M2‐2, RSVcps2 and LID/∆M2‐2 /1030s are the promising vaccine candidates for infants and children. The 60 µg of nanoparticle F vaccine candidate with adjuvant and Ad26.RSV.preF is two promising candidates for elderly adults. There is no promising vaccine for maternal vaccination at the present time.

## CONFLICT OF INTEREST

Professor Robert Booy: I work with all major manufacturers of vaccines in an advisory capacity, as a researcher on vaccines, as a presenter of academic information at conferences and also occasionally as an Advisory Board member. I receive support to travel and attend such conferences and meetings from the various pharmaceutical companies. On occasions, I also receive an honorarium which is paid direct to my institution. Any funding received is directed to a research account at The University of Sydney and is not personally accepted by myself. Other authors have no conflicts of interest.

## DATA AVAILABILITY STATEMENT

All data used in this study are included with in the article and supplementary file.

## AUTHOR CONTRIBUTION

**Jing Shan:** Data curation (lead); Formal analysis (lead); Investigation (equal); Methodology (equal); Software (equal); Writing‐original draft (lead). **Robert Booy:** Conceptualization (equal); Supervision (lead); Writing‐review & editing (equal). **Philip N. Britton:** Methodology (equal); Supervision (equal); Writing‐review & editing (equal). **Catherine L. King:** Data curation (equal); Resources (equal); Supervision (equal); Writing‐review & editing (equal).

### PEER REVIEW

The peer review history for this article is available at https://publons.com/publon/10.1111/irv.12850.

## Supporting information

Appendix S1
