# Supplementary material for: The immunogenicity and safety of respiratory syncytial virus vaccines in development: A systematic review
Source: Influenza Other Respir Viruses. 2021 Mar 25;15(4):539–51. doi: 10.1111/irv.12850 (PMC8189192; doi:10.1111/irv.12850)
Supplement: Supplementary file 1 — Appendix S1 [file IRV-15-539-s001.docx]

#### Quality assessment

Included clinical trials (n=38) from published journals were assessed by the first author (JS) using the Cochrane Collaboration’s risk of bias tool (Figure 2). The overall quality of the trials was good. All of them showed low risk of attrition and reporting bias.

24 trials reported low risk of randomisation sequence generation (1) (2) (3) (4) (5) (6) (7) (8) (9) (10) (19) (11) (12) (13) (14) (15) (30) (16) (17) (18) (19) (20) (21) (22), and 20 studies showed low risk of allocation concealment (3) (5) (23) (7) (9) (10) (24) (25) (12) (13) (14) (15) (26) (16) (17) (18) (19) (20) (21) (22). 27 trials blinded the participants and personnel (1) (27) (2) (3) (4) (5) (28) (6) (23) (7) (29) (8) (9) (10) (30) (31) (24) (32) (25) (12) (13) (14) (16) (17) (18) (21) (22), and 16 blinded the outcome assessment (1) (27) (2) (3) (4) (5) (28) (6) (23) (7) (29) (8) (9) (10) (24) (32).


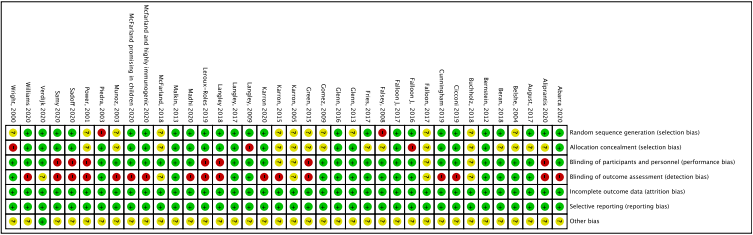


Figure 2 Summary of risk of bias in the trials from published journals

1 ***List of studies***: Abarca, 2020: (25); August, 2017: (1); Aliprantis, 2020: (11); Belshe, 2004: (27); Beran, 2018: (2) Bernstein, 2012: (3); Buchholz, 2018: (33); Cicconi, 2019: (12); Cunningham, 2019: (13); Falloon, 2017: (34); Falloon J, 2016: (4); Falloon J, 2017: (5) Falsey, 2008: (28).; Fries, 2017: (6); Glenn, 2013: (23); Glenn, 2016: (7); Gomez, 2009: (29); Green, 2015: (35); Karron, 2020: (14); Karron, 2005: (36); Karron, 2015: (37); Langley, 2009: (8); Langley, 2017 : (9); Langley, 2018: (15); Leroux-Roles, 2019: (26); Madhi, 2020: (16); Malkin, 2013: (10); McFarland and highly immunogenic, 2020: (17); McFarland promising in children, 2020: (18); McFarland, 2018: (30); Munoz, 2003: (31); Piedra, 2003: (24); Power, 2001: (38); Sadoff, 2020: (19); Samy, 2020: (20); Verdijk, 2020: (21); Williams, 2020: (22); Wright, 2000: (32). 2 The risk of bias summary provides a judgement about each risk of bias item for each published, included study. A green circle (with a plus mark) indicates low risk of bias. A red circle (with a minus mark) indicates high risk of bias. A yellow circle (with question mark) represents unclear risk of bias.3 Cochrane risk of bias assessment: low = average of 5/7 domains assessed as low risk of bias, high = average of 5/7 domains assessed as high risk of bias, and unclear for scores in between (39).

1. August A, Glenn GM, Kpamegan E, Hickman SP, Jani D, Lu H, et al. A Phase 2 randomized, observer-blind, placebo-controlled, dose-ranging trial of aluminum-adjuvanted respiratory syncytial virus F particle vaccine formulations in healthy women of childbearing age. Vaccine. 2017;35(30):3749-59.

2. Beran J, Lickliter JD, Schwarz TF, Johnson C, Chu L, Domachowske JB, et al. Safety and Immunogenicity of 3 Formulations of an Investigational Respiratory Syncytial Virus Vaccine in Nonpregnant Women: Results from 2 Phase 2 Trials. Journal of Infectious Diseases. 2018;217(10):1616-25.

3. Bernstein DI, Malkin E, Abughali N, Falloon J, Yi T, Dubovsky F. Phase 1 study of the safety and immunogenicity of a live, attenuated respiratory syncytial virus and parainfluenza virus type 3 vaccine in seronegative children. Pediatric infectious disease journal. 2012;31(2):109‐14.

4. Falloon J, Ji F, Curtis C, Bart S, Sheldon E, Krieger D, et al. A phase 1a, first-in-human, randomized study of a respiratory syncytial virus F protein vaccine with and without a toll-like receptor-4 agonist and stable emulsion adjuvant. Vaccine. 2016;34(25):2847-54.

5. Falloon J, Yu J, Esser MT, Villafana T, Yu L, Dubovsky F, et al. An Adjuvanted, Postfusion F Protein-Based Vaccine Did Not Prevent Respiratory Syncytial Virus Illness in Older Adults. Journal of Infectious Diseases. 2017;216(11):1362-70.

6. Fries L, Shinde V, Stoddard JJ, Thomas DN, Kpamegan E, Lu H, et al. Immunogenicity and safety of a respiratory syncytial virus fusion protein (RSV F) nanoparticle vaccine in older adults. Immunity & ageing. 2017;14(1) (no pagination).

7. Glenn GM, Fries LF, Thomas DN, Smith G, Kpamegan E, Lu H, et al. A Randomized, Blinded, Controlled, Dose-Ranging Study of a Respiratory Syncytial Virus Recombinant Fusion (F) Nanoparticle Vaccine in Healthy Women of Childbearing Age. Journal of Infectious Diseases. 2016;213(3):411-22.

8. Langley JM, Sales V, McGeer A, Guasparini R, Predy G, Meekison W, et al. A dose-ranging study of a subunit Respiratory Syncytial Virus subtype A vaccine with and without aluminum phosphate adjuvantation in adults > or =65 years of age. Vaccine. 2009;27(42):5913-9.

9. Langley JM, Aggarwal N, Toma A, Halperin SA, McNeil SA, Fissette L, et al. A Randomized, Controlled, Observer-Blinded Phase 1 Study of the Safety and Immunogenicity of a Respiratory Syncytial Virus Vaccine With or Without Alum Adjuvant. Journal of Infectious Diseases. 2017;215(1):24-33.

10. Malkin E, Yogev R, Abughali N, Sliman J, Wang CK, Zuo F. Safety and immunogenicity of a live attenuated RSV vaccine in healthy RSV-seronegative children 5 to 24 months of age. PloS one. 2013;8(10):e77104.

11. Aliprantis AO, Shaw CA, Griffin P, Farinola N, Railkar RA, Cao X, et al. A phase 1, randomized, placebo-controlled study to evaluate the safety and immunogenicity of an mRNA-based RSV prefusion F protein vaccine in healthy younger and older adults. Human Vaccines and Immunotherapeutics. 2020.

12. Cicconi P, Jones C, Sarkar E, Reyes LS, Klenerman P, de Lara C, et al. First-in-human randomized study to assess the safety and immunogenicity of an investigational respiratory syncytial virus (RSV) vaccine based on ChAd155 viral vector expressing RSV viral proteins F, N and M2-1 in healthy adults. Clinical infectious diseases. 2019.

13. Cunningham CK, Karron R, Muresan P, McFarland EJ, Perlowski C, Libous J, et al. Live-attenuated respiratory syncytial virus vaccine with deletion of RNA synthesis regulatory protein M2-2 and cold passage mutations is overattenuated. Open Forum Infectious Diseases. 2019;6 (6) (no pagination)(ofz212).

14. Karron RA, Luongo C, Mateo JS, Wanionek K, Collins PL, Buchholz UJ. Safety and immunogenicity of the respiratory syncytial virus vaccine RSV/DELTANS2/DELTA1313/I1314L in RSVSeronegative children. Journal of Infectious Diseases. 2020;222(1):82-91.

15. Langley JM, Macdonald LD, Weir GM, Mackinnon-Cameron D, Ye L, McNeil S, et al. A respiratory syncytial virus vaccine based on the small hydrophobic protein ectodomain presented with a novel lipid-based formulation is highly immunogenic and safe in adults: A first-in-humans study. Journal of Infectious Diseases. 2018;218(3):378-87.

16. Madhi SA, Polack FP, Piedra PA, Munoz FM, Trenholme AA, Simoes EAF, et al. Respiratory syncytial virus vaccination during pregnancy and effects in infants. New England Journal of Medicine. 2020;383(5):426-39.

17. McFarland EJ, Karron RA, Muresan P, Cunningham CK, Perlowski C, Libous J, et al. Live-Attenuated Respiratory Syncytial Virus Vaccine with M2-2 Deletion and with Small Hydrophobic Noncoding Region Is Highly Immunogenic in Children. Journal of Infectious Diseases. 2020;221(12):2050-9.

18. McFarland EJ, Karron RA, Muresan P, Cunningham CK, Libous J, Perlowski C, et al. Live Respiratory Syncytial Virus Attenuated by M2-2 Deletion and Stabilized Temperature Sensitivity Mutation 1030s Is a Promising Vaccine Candidate in Children. Journal of Infectious Diseases. 2020;221(4):534-43.

19. Sadoff J, De Paepe E, Haazen W, Omoruyi E, Bastian AR, Comeaux C, et al. Safety and Immunogenicity of the Ad26.RSV.preF Investigational Vaccine Coadministered With an Influenza Vaccine in Older Adults. The Journal of infectious diseases. 2020;26.

20. Samy N, Reichhardt D, Schmidt D, Chen LM, Silbernagl G, Vidojkovic S, et al. Safety and immunogenicity of novel modified vaccinia Ankara-vectored RSV vaccine: a randomized phase I clinical trial. Vaccine. 2020;38(11):2608‐19.

21. Verdijk P, van der Plas JL, van Brummelen EMJ, Jeeninga RE, de Haan CAM, Roestenberg M, et al. First-in-human administration of a live-attenuated RSV vaccine lacking the G-protein assessing safety, tolerability, shedding and immunogenicity: a randomized controlled trial. Vaccine. 2020;38(39):6088-95.

22. Williams K, Bastian AR, Feldman RA, Omoruyi E, de Paepe E, Hendriks J, et al. Phase 1 safety and immunogenicity study of a respiratory syncytial virus vaccine with an adenovirus 26 vector encoding prefusion F (Ad26.RSV.preF) in adults aged >=60 years. Journal of Infectious Diseases. 2020;222(6):979-88.

23. Glenn GM, Smith G, Fries L, Raghunandan R, Lu H, Zhou B, et al. Safety and immunogenicity of a Sf9 insect cell-derived respiratory syncytial virus fusion protein nanoparticle vaccine. Vaccine. 2013;31(3):524-32.

24. Piedra PA, Cron SG, Jewell A, Hamblett N, McBride R, Palacio MA, et al. Immunogenicity of a new purified fusion protein vaccine to respiratory syncytial virus: A multi-center trial in children with cystic fibrosis. Vaccine. 2003;21(19-20):2448-60.

25. Abarca K, Rey-Jurado E, Munoz-Durango N, Vazquez Y, Soto JA, Galvez NMS, et al. Safety and immunogenicity evaluation of recombinant BCG vaccine against respiratory syncytial virus in a randomized, double-blind, placebo-controlled phase I clinical trial. EClinicalMedicine. 2020;27 (no pagination)(100517).

26. Leroux-Roels G, De Boever F, Maes C, Nguyen TL, Baker S, Gonzalez Lopez A. Safety and immunogenicity of a respiratory syncytial virus fusion glycoprotein F subunit vaccine in healthy adults: Results of a phase 1, randomized, observer-blind, controlled, dosage-escalation study. Vaccine. 2019;37(20):2694-703.

27. Belshe RB, Newman FK, Anderson EL, Wright PF, Karron RA, Tollefson S, et al. Evaluation of combined live, attenuated respiratory syncytial virus and parainfluenza 3 virus vaccines in infants and young children. Journal of Infectious Diseases. 2004;190(12):2096-103.

28. Falsey AR, Walsh EE, Capellan J, Gravenstein S, Zambon M, Yau E, et al. Comparison of the safety and immunogenicity of 2 respiratory syncytial virus (rsv) vaccines--nonadjuvanted vaccine or vaccine adjuvanted with alum--given concomitantly with influenza vaccine to high-risk elderly individuals. Journal of infectious diseases. 2008;198(9):1317‐26.

29. Gomez M, Mufson MA, Dubovsky F, Knightly C, Zeng W, Losonsky G. Phase-I study MEDI-534, of a live, attenuated intranasal vaccine against respiratory syncytial virus and parainfluenza-3 virus in seropositive children. Pediatric Infectious Disease Journal. 2009;28(7):655-8.

30. McFarland EJ, Karron RA, Muresan P, Cunningham CK, Valentine ME, Perlowski C, et al. Live-attenuated respiratory syncytial virus vaccine candidate with deletion of RNA synthesis regulatory protein M2-2 is highly immunogenic in children. Journal of Infectious Diseases. 2018;217(9):1347-55.

31. Munoz FM, Piedra PA, Glezen WP. Safety and immunogenicity of respiratory syncytial virus purified fusion protein-2 vaccine in pregnant women. Vaccine. 2003;21(24):3465-7.

32. Wright PF, Karron RA, Belshe RB, Thompson J, Crowe JE, Jr., Boyce TG, et al. Evaluation of a live, cold-passaged, temperature-sensitive, respiratory syncytial virus vaccine candidate in infancy. Journal of Infectious Diseases. 2000;182(5):1331-42.

33. Buchholz UJ, Cunningham CK, Muresan P, Gnanashanmugam D, Sato P, Siberry GK, et al. Live respiratory syncytial virus (RSV) vaccine candidate containing stabilized temperature-sensitivity mutations is highly attenuated in RSV-seronegative infants and children. Journal of Infectious Diseases. 2018;217(9):1338-46.

34. Falloon J, Talbot HK, Curtis C, Ervin J, Krieger D, Dubovsky F, et al. Dose Selection for an Adjuvanted Respiratory Syncytial Virus F Protein Vaccine for Older Adults Based on Humoral and Cellular Immune Responses. Clinical & Vaccine Immunology: CVI. 2017;24(9).

35. Green CA, Scarselli E, Sande CJ, Thompson AJ, De Lara CM, Taylor KS, et al. Chimpanzee adenovirus- and MVA-vectored respiratory syncytial virus vaccine is safe and immunogenic in adults. Science Translational Medicine. 2015;7(300).

36. Karron RA, Wright PF, Belshe RB, Thumar B, Casey R, Newman F, et al. Identification of a recombinant live attenuated respiratory syncytial virus vaccine candidate that is highly attenuated in infants. Journal of infectious diseases. 2005;191(7):1093‐104.

37. Karron RA, Luongo C, Thumar B, Loehr KM, Englund JA, Collins PL, et al. A gene deletion that up-regulates viral gene expression yields an attenuated RSV vaccine with improved antibody responses in children. Science Translational Medicine. 2015;7(312):312ra175.

38. Power UF, Nguyen TN, Rietveld E, De Swart RL, Groen J, Osterhaus ADME, et al. Safety and immunogenicity of a novel recombinant subunit respiratory syncytial virus vaccine (BBG2Na) in healthy young adults. Journal of Infectious Diseases. 2001;184(11):1456-60.

39. Higgins JP, Altman DG, Gotzsche PC, Juni P, Moher D, Oxman AD, et al. The Cochrane Collaboration's tool for assessing risk of bias in randomised trials. Bmj. 2011;343:d5928.
